# Safety study of cannabidiol products in healthy dogs

**DOI:** 10.3389/fvets.2024.1349590

**Published:** 2024-03-01

**Authors:** William Bookout, Margitta Dziwenka, Kaiti Valm, Jennifer Kovacs-Nolan

**Affiliations:** ^1^National Animal Supplement Council (NASC), Sun City West, AZ, United States; ^2^Nutrasource Pharmaceutical and Nutraceutical Services, Guelph, ON, Canada

**Keywords:** cannabinoid, canine, CBD, CBDA, CBG, cannabis, hemp, NASC

## Abstract

The tolerability of different cannabinoids given orally to dogs was evaluated in a randomized, non-blinded, negative controlled, parallel design 90-day repeat dose study with a 14-day recovery period. Healthy beagles (16 males and 16 females) were randomized into four treatment groups and treated with either medium chain triglyceride oil as the control or one of the following: broad spectrum cannabidiol, broad spectrum cannabidiol with cannabigerol, or broad spectrum cannabidiol with cannabidiolic acid at 5 mg total cannabinoids/kg body weight/day. Animals were observed daily with detailed clinical examinations conducted weekly. Animals were monitored for an additional 2 weeks after dosing. Body weights, food consumption and clinical pathology evaluations were included in the study. Cannabinoids were well tolerated when healthy male and female beagles were dosed for 90 consecutive days. Annual post-market surveillance data for hemp-derived supplement products sold for use in dogs from 2010 to 2023 (partial year) shows that the rate per 1 million administrations sold is 2.10 for adverse events and 0.01 for serious adverse events. Based on the results of this study, other published studies, and data from extensive post-market surveillance, hemp-derived cannabinoids are well tolerated in healthy dogs at a dose of 5 mg/kg body weight/day.

## 1 Introduction

Cannabinoid products derived from *Cannabis sativa* L., specifically hemp (defined in U.S. Code of Federal Regulations Title 7 Part 1437.3 “Hemp” as *C. sativa* containing <0.3% Δ9-tetrahydrocannabinol (THC)), are increasing in use for both humans and their pets. Consumer and veterinary surveys indicate use in pets is notable and likely to grow and consumer understanding of the products they are giving their pets is low. Alvarenga et al. (1) collected data from 1,238 survey participants (mostly in the US) via a website that pools participants specifically for online research. They reported that 28.8% (*n* = 356) of respondents indicated they currently give or had given their pet cannabidiol (CBD) or cannabis product and 51.4% (*n* = 882) indicated they would be interested in giving their pet a CBD or cannabis product. Of the respondents who were currently giving or had given a supplement, CBD isolate was the most commonly identified product (100% CBD, 25.8%, *n* = 92). Broad spectrum (described as 0% THC, 16.6%, *n* = 59) and full spectrum (includes THC, 15.2%, *n* = 54) were also commonly used. However, many survey respondents did not know the purity or composition (42.4%, *n* = 151) (1). There are gaps in information for veterinarians as well and the veterinary community is not fully equipped to council clients on CBD use for their pets. In an anonymous survey of 2,130 US veterinarians in 2018, approximately one third (35%) said they “did not know much” about the therapeutic effects of hemp/CBD, and 43.7% of respondents indicated they “did not know much” about the toxic effects of hemp/CBD products. The majority (86.4%) of respondents agreed or strongly agreed that therapeutic use and toxicity of hemp/CBD should be researched (2).

The body of evidence for safety of cannabinoid product use in dogs, from both consumer reports and scientific studies, is small but growing. Conversely, the risk of cannabis toxicosis in pets increases with the increasing availability of consumer products, therefore further research on the safety and effectiveness of cannabis products is warranted (3, 4). Information regarding the safety of hemp extracts and isolated hemp cannabinoids from preclinical studies in rodents can be utilized to determine the safety of these extracts, however additional information is required from studies conducted in dogs to adequately determine the safe doses to utilize (5–7). Pharmacokinetic data from recent studies in dogs is available for broad spectrum CBD (8, 9), purified CBD (10), CBD/ cannabidiolic acid (CBDA) (11–13), and cannabigerol (CBG) with cannabigerolic acid (CBGA) (14), as well as delivery methods other than oral (15–17), CBD with THC in a 1:20 THC: CBD ratio (18), and Sativex® (19).

Duration of use, product form, and vehicle (e.g., oil-based extracts) for delivery of cannabinoids have been explored. Alvarenga et al. (9) completed a long-term (36 week) study of broad-spectrum CBD (95% of cannabinoid profile) in a medium chain triglyceride (MCT) vehicle. The authors reported that CBD accumulated in dogs over time as the half-life tripled by the 18-week mark and stayed at that level until 36 weeks, and this effect was proportional to the dose (9). Deabold et al. (8) gave doses of 2 mg CBD/CBDA mix/kg bodyweight (bw)/day to fasted dogs (*n* = 6) over a 12-week period in a chew format. The authors noted that delivery in a chew resulted in a shorter retention time and half-life than an infused oil (8). Wakshlag et al. (11) determined the pharmacokinetics of three different forms of an infused oil containing equal amounts CBD and CBDA and small amounts of THC and tetrahydrocannabinolic acid (THCA). They determined that a vehicle of 25% sunflower lecithin increased the absorption of CBDA and THCA, demonstrating that the vehicle has the potential to affect the safety profile (11).

Oral dosing with cannabinoids in dogs is generally well tolerated. Di Salvo et al. (20) summarized 19 tolerability studies with CBD or CBD/CBDA. Of the two studies that extended beyond 12 weeks duration, one used a CBD-only distillate at approximately 4 mg/kg bw/day and the other used a highly purified CBD (Epidiolex) at up to 100 mg/kg bw/day. Five studies of 12 weeks duration using CBD or CBD/CBDA products were also summarized. Common side effects noted were increase in ALP activity, GI symptoms, somnolence, and ataxia. No serious side effects were noted (20).

This study in healthy male and female beagles given a daily treatment dose for 90 consecutive days evaluates broad spectrum CBD, broad spectrum CBD with CBDA, and is the first to our knowledge to include broad spectrum CBD with CBG in a long-term tolerability study. Given the volume of consumer products sold annually, data from well controlled studies with defined safety endpoints and doses relative to industry use are imperative to understand the risk associated with cannabinoid use in dogs. It is expected that doses of 5 mg/kg bw/day will have no adverse effects in healthy beagles. The current study adds to the available literature evaluating the tolerability of broad-spectrum cannabinoid products in healthy dogs in a fed state.

## 2 Materials and methods

### 2.1 Study conduct

This study was conducted by ClinVet USA LLC, an Association for Assessment and Accreditation of Laboratory Animal Care accredited facility which conforms to the guidelines set forth in the National Research Council Guide for the Care and Use of Laboratory Animals (8th Edition, 2011). All procedures were designed in accordance with the principles of the USDA Animal Welfare Act (7 USC § 2,131–2,159) as well as U.S. Code of Federal Regulations Title 9, Part 3. The study protocol was approved by the Institutional Animal Care and Use Committee.

### 2.2 Animals

Thirty-two intact healthy beagle dogs (16 males and 16 females), with an average age of 18.4 months ± 6.7 (range 11 to 32 months) and weighing an average of 9.9 kg ± 1.2 (range 8.2 to 12.8 kg) at study start, were included in this study. Female dogs were checked for pregnancy prior to inclusion in the study. See section 3.4 Assessment for further information on health assessments. All dogs were housed individually in stainless steel cages, which were cleaned daily and sanitized at least bi-weekly. An acclimation period of 14 days in the housing room was provided. All animals had access to visual, auditory, and olfactory contact during the study. Dogs were exercised with their respective treatment groups and sexes outside of their cages during daily husbandry duties. A 12-h light/dark cycle was maintained throughout the study. All dogs were fed Parable Agriculture Custom 30–22 Dog Food, from Pro-Pet, LLC (dry food) in a daily ration with *ad libitum* water. Animals were dosed daily for 90 days and were then observed for an additional 14 days without dosing. At the end of the study, the animals were returned to the testing facility colony.

### 2.3 Study design

This study was a randomized, non-blinded, negative controlled, parallel group design. The dogs were randomized by block design into 4 groups. Four sex-balanced groups were created by ranking females (*n* = 16) by decreasing weight, males (*n* = 16) by increasing weight, and blocking the animals into 8 groups of 4 dogs. Within blocks, the dogs were allocated randomly to the treatment groups. Each treatment group was given one daily oral dose of: broad spectrum CBD (test article (TA) 1; group 2), CBD + CBG combination (TA2; group 3), CBD + CBDA combination (TA3; group 4), or MCT oil (Control; group 1) for 90 days. The dogs were fasted overnight after removal of any remaining daily ration and received a normal ration in the morning prior to dosing. Dogs were dosed when in a fed state and doses were delivered orally via syringe.

The daily dose of the test materials was 5 mg of total cannabinoids/kg bw and the volume of the control MCT oil was correlated with the volume dosed in the treatment groups. TA1 was CBD of 80–90% purity, manufactured by Open Book Extracts, Roxboro, NC. TA2 was CBD + CBG in a 1:2 ratio, manufactured by Open Book Extracts. TA3 was CBD + CBDA in a 1:1 ratio, manufactured by KND Labs, Lakewood, CO (Table 1). The control article was MCT oil sourced from coconut or palm kernel, manufactured by Jedwards International, Inc., Braintree, MA.

**Table 1:** Composition of cannabinoid test articles (TA) used in the 90-day repeat dose study.

Test article	TA1: CBD	TA2: CBD + CBG	TA3: CBD + CBDA
Lot #	BFG-000030-220505	BFG-000031-220505	KND 1:1-CBD/A-MCT-595
CBD (mg/g)	37.01	13.52	17.1100
CBG (mg/g)	0.99	24.56	0.7356
CBDA (mg/g)	ND	ND	18.5308
CBDV (mg/g)	0.13	0.08	ND
CBN (mg/g)	ND	ND	0.5885
CBGa (mg/g)	ND	ND	ND
CBC (mg/g)	0.29	0.11	0.5347
THC (mg/g)	ND	ND	ND
Total cannabinoids (mg/g)	38.42	38.26	35.545
Total terpenes (mg/g)	2.080	0.740	0.0125
Residual solvents	ND	ND	ND*
Heavy metals (μg/g)	ND	ND	<LOQ (0.05)
Pesticides	ND	N1	ND
Microbials	ND	ND	ND

CBC, Cannabichromene; CBD, Cannabidiol; CBDA, Cannabidiolic acid; CBDV, Cannabidivarin; CBG, Cannabigerol; CBGa, Cannabigerolic acid; CBN, Cannabinol; LOQ, Limit of Quantitation; ND, Not detected; THC, Tetrahydrocannabinol.

*With the exception of acetonitrile which was present at 73.0 μg/g (Limit = 5,000 μg/g).

### 2.4 Assessments

Clinical examinations were performed on all animals during acclimatization (between day −14 and − 1), and days 14, 28, 56, 90 and 104. Clinical examination included but was not limited to vital signs, mucous membranes, eyes, motility, lymph nodes, abdominal palpations, thoracic auscultation, skin condition, behavior, reproductive system, respiratory, cardiac, gastrointestinal, and urinary systems. All animals were also observed twice daily for habitus, color of urine, color and consistency of feces, salivation, vomiting, skin lesions, and obvious change in general condition. Body weights were measured on days −8, −1, and weekly throughout the study. Adverse events (AE) were considered to be any observation that was unfavorable or unintended and occurred anytime during the dosing period (after day 0). Serious adverse events (SAE) were defined as AE that were fatal or life threatening.

Food consumption was determined by weighing food prior to and after feeding each animal daily from day −7 through the end of the study. Blood samples were collected into serum separator tubes (2 mL whole blood), sodium citrate tube (2.7 mL whole blood), and EDTA tube (1.0 mL whole blood) from fasted animals on days −9, 14, 28, 56, 90 and 104 for clinical pathology. Serum from the separator tube was allowed to sit at room temperature for 1 h prior to separation. Plasma from the sodium citrate tube was separated after centrifuging for 10 min at room temperature, then plasma was separated and frozen at −60°C to −90°C before transport to the laboratory. The EDTA tube was not processed. Analyses included hematology, serum chemistry, and coagulation parameters. Hematology parameters were erythrocytes, hemoglobin, leukocytes, MCH, MCHC, MCV, PCV, and platelet count. Serum chemistry parameters were ALT, albumin, ALP, amylase, AST, calcium, chloride, cholesterol, creatine kinase, creatinine, globulin, GGT, glucose, LDH, magnesium, phosphate, potassium, sodium, total protein, and urea nitrogen. Serum chemistry analyses were performed using a Roche Cobas c501 (Roche Diagnostics, Indianapolis, IN, USA) and hematology analyses were completed with a Siemens Advia 2120i (Siemens Medical Solutions USA, Inc. Malvern, PA USA). Coagulation parameters were prothrombin time, fibrinogen, and activated thromboplastin time. Coagulation parameters were analyzed using a Diagnostica Stago STA Compact Max (Diagnostica Stago S.A.S., France). Urine was collected via passive collection in the morning on days −8 /−7, 28, 90 and 104. Urine samples were analyzed for turbidity, specific gravity, pH, protein, glucose, ketones, blood, and bilirubin using a Siemens Clinitek Advantus (Siemens Medical Solutions USA, Inc. Malvern, PA USA). All samples were sent for analysis on the day of collection and analyzed within 1 day.

### 2.5 Statistical analysis

Statistical analysis procedures were based on International Cooperation on Harmonization of Technical Requirements for Registration of Veterinary Medicinal Products (VICH) Guideline GL43: Target Animal Safety for Veterinary Pharmaceutical Products. Baseline data was considered the last non-missing value for each parameter prior to dosing. Individual hematology and serum chemistry parameters were reported with descriptive statistics: mean, SD, coefficient of variation, geometric mean, median, minimum, maximum, and number of observations (n) in that treatment group. For identifying parameter values that warrant further clinical review, a reference range was defined as the minimum and maximum values for each parameter at baseline across all groups of dogs in the current study. Because the primary intent of this study was to evaluate tolerability of each formulation, the magnitude of changes from baseline (CFB) to each of the post-administration days were calculated for hematology and serum chemistry parameters. If a parameter for any individual on any post-administration day fell outside the reference range, the CFB for the treatment group was compared to the CFB for the control group, and the CFB within the treatment group was checked for significance. If all the above were statistically significant, further clinical review was completed. Within each treatment, post-administration values were compared to baseline by means of ANOVA with animal and observation time as effects for all laboratory parameters. Between-treatment comparisons of CFB on each post-administration day were performed using a linear mixed model with TA administration as fixed effect and randomization block as random effect. The results of all other measured or observed parameters (clinical examinations, general observations, bw, and food consumption) are reported descriptively and tabulated when appropriate. The level of significance for all formal tests was set at 5% and all tests were two-sided. SAS version 9.4 was used for all statistical analyses.

## 3 Results

All animals completed the study, and no somnolence, AE or SAE were reported during the study. Sporadic hypersalivation was reported in some animals in the CBD + CBG and CBD + CBDA treatments but these were not deemed to be an AE. In all groups, abnormal and incidental findings were reported in some animals during the daily visual examinations or the more detailed clinical examinations. These were deemed to be unrelated to test or control article exposure and did not negatively impact the results of the study. The most common abnormal observation was diarrhea. There were no statistically significant differences in bw between control and treatment groups at the start of the study and all groups had a higher mean bw on day 104 as compared to the baseline values at the start of the study (Supplementary Table S1). The majority of animals consumed their daily ration each day and differences in mean food consumption within all groups were sporadic and not considered to be an adverse finding (Supplementary Table S2). Clinical pathology data (including hematology and serum chemistry) is presented as mean ± SD for each time point tested. There were statistically significant within-treatment group changes reported in some hematology (Supplementary Table S3) and clinical chemistry (Supplementary Table S4) parameters evaluated as compared to the baseline value. The majority of the changes reported in the hematology parameters evaluated were either transient, had no concurrent clinical signs or correlating changes in other related clinical pathology parameters, or were within reference ranges and were not considered to be a clinically relevant adverse effect of test material treatment. On day 14, one animal receiving the CBD + CBDA treatment had a hemoglobin value slightly below the reference range and on day 56, one animal in the same treatment group had a WBC value which was slightly above the reference range. These changes were not considered clinically relevant due to the transient and /or isolated nature of the changes.

At each time point, the CFB was calculated and comparisons between control and treatment groups with respect to CFB were carried out to determine significance. Statistically significant changes in the mean CFB values of a number of clinical chemistry parameters were reported in the cannabinoid treatment groups compared to the mean CFB values of the control group (Supplementary Table S5). These changes were of a low magnitude and/or transient and/or were within the reference ranges and/or had no correlating changes in related parameters and were therefore determined to be of no clinical relevance.

Some of the changes in clinical chemistry parameters in individual animals were outside of the reference ranges and are discussed. On day 14, one animal in the CBD + CBDA treatment group had a urea value which was slightly below the reference range. One animal in the CBD + CBG treatment group had a potassium value which was slightly below the reference range on study days 14 and 28, while one animal in the CBD + CBDA treatment group had a potassium value which was slightly lower than the reference range on study day 28 only. One animal in the CBD + CBDA treatment group had an iron value lower than the reference range on study day 28 and a sodium level which was slightly above the reference range on study day 56. Two animals showed an increased chloride value which was above the reference range on study day 56 in the CBD + CBDA treatment group. Sodium was elevated to levels above the reference range in one animal in the CBD + CBDA treatment group on study day 56 and in a different animal in the same group on study day 90 as well as in one animal in the CBD + CBG treatment group on study day 90 as well. On study day 104, creatinine kinase values were found to be sporadically elevated above the reference range including in 2 animals in the control group. On study day 104, albumin in one animal in the CBD + CBG treatment group was slightly below the reference range. Given the low magnitude of the changes seen in the clinical chemistry parameters, the transient nature and lack of corresponding clinical or clinicopathological changes, the changes described were considered to be of no clinical relevance.

Changes in ALT, ALP and GGT were reported during the study. For the treatment groups, the CFB was compared to the CFB for the control group for the specific study day (Table 2). The only statistically significant change in GGT CFB values was reported on study day 28 in the CBD treatment group which decreased less than the concurrent controls. Mean CFB for ALP values showed an increase from baseline and were significantly higher in the CBD treatment group on study days 28, 56 and 90 and the CBD + CBDA treatment group on study day 56 as compared to the mean CFB values in the control group, which decreased from baseline. Within these groups, all values for ALP were within the laboratory reference ranges (range 7–115 U/L) with the exception of one animal in the CBD treatment group on study days 28 (314 U/L), 56 (227 U/L) and 90 (205 U/L) and one animal in the CBD + CBDA treatment group on study day 56 (123 U/L) in which the values were above the reference range. Of the values which were outside of the upper reference range for the single animal in the CBD treatment group, two of the three were below a twofold increase and the remaining value peaked at 314 U/L on study day 28 but then decreased in each of the following evaluations and was within the reference range following the 14-day recovery period. The value for the only other animal with a value above the reference range occurred on study day 56 and was below a twofold increase, and the values were within the reference range at the next evaluation on study day 90. Mean CFB ALT values for all treatment groups decreased from baseline, whereas in the control group, values decreased from baseline on day 14 and increased from baseline at all other time points. This resulted in a significant CFB in the CBD + CBG treatment group on study days 28, 56 and 104, as compared to the control group, however none of the mean values were outside of the reference range at any time (Supplementary Table S4).

**Table 2:** Baseline and mean change from baseline (CFB) serum ALT, ALP, and GGT results for healthy beagles treated orally with medium chain triglyceride (MCT) oil (Control; *n* = 8) or 5 mg/kg body weight/day of CBD (*n* = 8), CBD + CBG (*n* = 8) or CBD + CBDA (*n* = 8) for 90 days, followed by 14 days without dosing.

Study day	Control	CBD	CBD + CBG	CBD + CBDA
**ALT (U/L)**				
−9 (Baseline)	33.3 ± 9.7	39.8 ± 13.8	42.4 ± 12.8	40.6 ± 15.1
	Change from baseline			
14	−3.1 ± 9.0	−3.5 ± 12.0	−8.9 ± 6.6	−8.8 ± 6.0
28	1.4 ± 3.5	−4.5 ± 11.0	−7.0 ± 5.5*	−3.9 ± 3.4
56	0.6 ± 5.0	−5.1 ± 10.2	−7.3 ± 8.3*	−6.3 ± 5.1
90	4.0 ± 3.8	−3.6 ± 10.0*	−2.9 ± 8.9	−5.0 ± 5.6*
104	3.3 ± 2.9	2.3 ± 12.2	−7.1 ± 9.8*	−2.6 ± 5.2
**ALP (U/L)**				
−9 (Baseline)	23.9 ± 10.6	31.1 ± 10.5	28.8 ± 9.6	31.1 ± 7.0
	Change from baseline			
14	−2.9 ± 4.3	21.5 ± 40.5	0.3 ± 3.1	22.0 ± 45.1
28	−1.4 ± 2.3	56.5 ± 89.5*	4.8 ± 5.7	31.6 ± 37.6
56	−2.3 ± 3.7	41.8 ± 58.5*	6.1 ± 8.2	34.8 ± 26.4*
90	−0.6 ± 3.3	38.5 ± 52.1*	5.9 ± 9.4	19.6 ± 22.4
104	1.3 ± 3.7	10.9 ± 17.1	9.6 ± 22.9	6.6 ± 8.9
**GGT (U/L)**				
−9 (Baseline)	2.5 ± 1.3	2.0 ± 0.9	2.5 ± 0.9	2.4 ± 1.4
	Change from baseline			
14	−0.5 ± 1.3	0.1 ± 1.7	0.4 ± 0.7	−0.3 ± 1.5
28	−1.8 ± 1.2	−0.1 ± 1.0*	−0.6 ± 1.2	−0.6 ± 1.5
56	0.0 ± 0.9	0.9 ± 1.5	0.4 ± 1.4	0.3 ± 1.7
90	0.3 ± 0.9	0.9 ± 0.8	0.4 ± 0.9	0.3 ± 2.1
104	0.1 ± 1.1	0.6 ± 1.4	−0.8 ± 0.9	−0.6 ± 1.4

Data are reported as mean ± SD for baseline and mean change from baseline ± SD of serum chemistry parameters on each assessment day. Between-group comparisons with respect to CFB were carried out to compare control and cannabinoid groups to determine significance.

*CFB is significantly different from control group on the same assessment day (row) (*p* < 0.05).

U/L, Units per liter.

There were statistically significant changes in some of the coagulation parameters evaluated however all measured values were within the reference ranges except for one animal in the CBD treatment group which had an elevated fibrinogen value which was deemed to be clinically irrelevant (Supplementary Table S6). Urine was collected prior to dosing and then on study days 28, 90 and 104 and no clinically relevant changes were reported in any treatment groups as compared to controls. Specific gravity and urine pH are summarized in Supplementary Table S7. No significant abnormalities were recorded for any urinalysis parameters evaluated. All animals were returned to the Test Site colony at the end of the study.

## 4 Discussion

In the current study, daily exposure to CBD, CBD + CBG and CBD + CBDA at 5 mg/kg bw of total cannabinoids for 90 consecutive days was well tolerated. The significant changes seen in some clinical pathology parameters were transient, within reference ranges, of low magnitude, present in a small number of animals or sporadic in nature and all were considered not to be clinically relevant. Biological variability is discussed in Flatland et al. where the authors concluded that a single clinical value needs to be interpreted within three aspects of variation – individual, group, and analytical method (21). In the current study, review for clinical relevance was determined using a reference range set by the baseline values of the animals in the study as previously described. If a parameter for any individual on any post-administration day fell outside this reference range, the CFB for the treatment group was compared to the CFB for the control group, and the CFB within the treatment group was checked for significance. If all the above were statistically significant, further clinical review was completed. Following this method, individual and group variation is accounted for via reference range determination and by placing emphasis on the CFB as indicative of a treatment-related change but only if the treatment group CFB was different from the control group CFB for any parameter. Analytical variation is not applicable in this study as all measurements were made under the same conditions as part of a research study, and not in a clinical setting where variation between equipment, staff, etc. could be notable.

The results from this study correlate with other studies conducted with CBD in healthy dogs which have concluded that CBD, CBG, and CBD with CBDA is well tolerated. Bradley et al. (22) conducted a randomized, placebo-controlled, blinded study with broad-spectrum CBD in healthy dogs. The CBD treated dogs received 4 mg/kg bw/day for 6 months without any adverse effects on health and wellbeing. A transient elevation in ALP was reported in approximately half of the CBD treated dogs which returned to baseline at the end of the 4-week recovery period. Bone ALP was evaluated to determine the tissue source of the ALP and was significantly elevated as compared to controls at the end of 26 weeks of treatment with a significant and strong positive correlation between ALP and bone ALP. Based on these and other results, the authors concluded that the increased ALP was not a clinically relevant biomarker of impaired liver health in healthy dogs following CBD treatment (22). Vaughn et al. (23) also evaluated the safety of CBD in healthy dogs in a 28-day repeat dose trial. In the randomized, blinded, placebo-controlled study, the healthy dogs received either a placebo or 1, 2, 4 or 12 mg CBD/kg bw/day which was well tolerated. All reported AE were mild and self-limiting and occurred in all groups, including the placebo group. Increased serum ALP above the upper reference limit was reported in the 2, 4 and 12 mg/kg bw/day groups which began to decrease after 2 weeks of dosing, but these animals did not have any concomitant increases in other hepatic markers. As with the current study, hypersalivation was seen with greater frequency in the CBD treated groups but this was not considered to be a SAE in either study (23). Deabold et al. (8) evaluated the safety and adverse effects of a CBD containing hemp product in healthy dogs over a 12-week dosing period. The dogs were given 2 mg CBD/kg bw/day and serum chemistry and hematology evaluations showed no clinically relevant changes during the study (8).

In a study by Amstutz et al. (14), CBG and CBGA was trialed in fed and fasted dogs (*n* = 6 intact male beagles) at 2 mg/kg bw twice daily for 2 weeks. The fasted state was tested initially for two-weeks, followed by a two-week washout and then treatment was given in the fed state for two-weeks. On the first day of treatment in both states, a 24-h pharmacokinetic analysis of serum cannabinoids was completed. The authors reported that there were no statistically significant differences in pharmacokinetic parameters between fed and fasted states, however, they note that the serum concentration of CBG tended to be higher in the fasted state. Serum ALP decreased in both fed and fasted states by week 2, which is contrary to other studies of cannabinoids. The authors suspect this may be related to differences in the effect of CBG and CBD on cytochrome P450 although no further evidence is discussed. The only AE reported was vomiting from one dog during the fasting phase with no other clinical symptoms. The authors concluded that CBG and CBGA at 2 mg/kg bw twice daily was well tolerated in fed and fasted healthy beagle dogs (14). In the current study, the test item contained CBD + CBG in a 1:2 ratio. Although not clinically relevant, the CBD and CBD/CBDA treatment groups each showed at least one measurement that was statistically different from the control group for ALP, whereas the CBD/CBG group did not differ from the control group.

Two studies evaluated CBD/CBDA mixed cannabinoid products. Tittle et al. (13) evaluated the pharmacokinetics of a CBD/CBDA extract that also included a low level of THC/THCA when dosed in oil verses a gel capsule. Beagles (7 male and one female) were dosed at 2 mg/kg bw twice daily with food. The initial treatment was the cannabinoid product in a capsule. Pharmacokinetic parameters were measured over 24 h on the first day of dosing and over a subsequent 7 days, followed by a 2-week washout period before the next treatment (cannabinoids in oil) and pharmacokinetic measurements for 7 days. No safety end points were assessed, however, AE noted were mild and included vomiting, diarrhea, licking, and head shaking. Vomiting and diarrhea was observed in three dogs during the washout period as well (13). Wakshlag et al. (11) evaluated a CBD/CBDA product that contained a small amount of THC and THCA, as well as measurable CBGA and cannabichromene. The intent of this study was to evaluate two different oil vehicles and a soft chew format with 2-week treatments followed by 3-week washout periods, resulting in a 12-week trial. Six intact female beagles were dosed at 2 mg/kg bw (oil) or 2.0–2.3 mg/kg bw (soft chew) of CBD/CBDA twice daily. Safety end points measured included ALP, AST, and ALT, albumin, total bilirubin, cholesterol, and glucose. No changes were observed in these parameter during treatment or between successive treatments, and no abnormalities in behavior or health were reported during the trial (11). A key difference in the test item for these studies compared to the current study was the presence of THC at a low level, however, like the current study, no AE related to administration of CBD/CBDA were reported.

Several studies evaluated the clinical efficacy of CBD and other cannabinoids in disease and behavioral conditions. The endpoints evaluated in these studies may not be specifically targeted towards safety, but they can provide some valuable tolerability information regardless. For example, studies have been conducted to evaluate the analgesic effect of CBD in dogs with spontaneous osteoarthritis at varying dose levels and durations, as well as in dogs who recently underwent orthopedic surgery. The dogs received up to 5 mg/kg bw orally for 4 weeks following surgery, which was shown to be well tolerated (12, 24–27). Treatment with CBD-CBDA was evaluated for efficacy on refractory epileptic seizures, intractable idiopathic epilepsy, atopic dermatitis, and immune response (28–31). The effect of CBD on behavioral conditions such as aggression towards animal shelter staff, separation anxiety and car travel, noise-induced fear, and voluntary activity was also evaluated (32–35). In all studies, few minor or zero AE were reported and no SAE that could be attributable to treatment were reported.

In the United States, “pet supplements,” also called Dosage Form Animal Health Products, are unapproved animal drugs and available to consumers either through State-level regulations or enforcement discretion by the FDA (3). Products containing CBD are sold in substantial numbers and post-market surveillance data supports the safety of cannabinoids given orally. In 2022, there were 274,129,622 administrations, in dogs, of hemp and hemp derivative products sold, as determined by the National Animal Supplement Council (NASC). The NASC is a 501(c) (6) non-profit trade association that represents most of the industry selling products containing hemp, hemp derived compounds as well as cannabinoids in the US. The NASC requires all member companies marketing products to enter product information, upload product labels and to report AE monthly through its Adverse Event Reporting System (NAERS™) which is a powerful tool for post-market surveillance. Individual companies are also required to record, report, and evaluate AE monthly. Both serious and non-serious AE are reported in the NAERS™ system. Each AE is evaluated and given a risk score using the NASC Adverse Event reporting form, which is also maintained in the NAERS™ system.

In the NAERS™ system, AE and SAE are defined as follows:

- **Adverse Event:** “An Adverse Event is a type of Complaint where a patient has suffered any negative physical effect or health problem that MAY be connected to or associate with use of the product.”
- **Serious Adverse Event:** “An Adverse Event with a transient incapacitating effect (i.e., rendering the animal unable to function normally for even a short period of time, such as with a seizure) or non-transient (i.e., permanent) health effect. Transient vomiting or diarrhea do not constitute Serious Adverse Events. A purported Serious Adverse Event requires follow-up with a veterinarian. A layperson diagnosis does not constitute a Serious Adverse Event.”

Data from each company is aggregated, statistically processed, and compiled into an Ingredient Risk Report which provides information relating the ingredient(s) to reported AE, both serious and non-serious. The event rates are reported based on the number of administrations in each container sold and unit data is updated quarterly.

Data from NASC Members’ products was also used in determining the dosing level used in the current study, 5 mg/kg bw of dogs, which is based on actual products currently in the marketplace. Based on the information from the Ingredient Risk Report, the straight mean and weighted mean doses for all hemp and hemp derivative products were determined to be 6.97 and 9.91 mg/kg bw. Comparatively, the straight mean and weighted mean doses for all CBD products were 0.83 mg/kg and 0.67 mg/kg (maximum 2.10 mg/kg). This provides important information that is difficult to ascertain from consumer surveys. Alvarenga et al. (1) reported that when asked about dose, survey respondents gave empiric answers of volume without concentration or missing a measuring unit, making analysis and reporting unfeasible (1).

The use of CBD in Dosage Form Animal Health Products has been growing; however, the safety of longer-term use has been questioned and deemed to be lacking (8, 22). A recent review of the current literature available for CBD use in dogs documented 19 tolerability studies, 10 pharmacokinetic studies with oral CBD products, seven clinical trials for efficacy in pain control, three for epilepsy, three for behavioral disorders, and three for skin diseases (20) The limitations of this body of evidence are that the number of types of extracts, the study population, and the duration of use are constrained by necessity. Post-market surveillance of AE and SAE in the NAERS™ database assists in the safety evaluation of CBD through real-world use data and supports the conclusion of the aforementioned studies that CBD products are well tolerated.

The information collected from the NAERS™ system report for all products containing hemp and hemp derived compounds shows that the overall report rate per million administrations sold from 2010 to 2023 (as of November 20th, 2023) for AE and SAE in dogs is 2.19 and 0.01, respectively, from over one billion administrations (Table 3). When limited to products specifying CBD, the total administrations in dogs for 2015–2023 (as of November 18, 2023) were 86,081,473, with AE and SAE rates of 1.61 and 0.02 per million administrations, respectively.

**Table 3:** National Animal Supplement Council (NASC) Ingredient Risk Report for hemp and hemp-derived compounds in dogs as of November 20, 2023.

Year	Adverse events (report rate/million administrations sold)	Serious adverse events (report rate/million administrations sold)	Administrations sold^a^
2010	0.00	0.00	25,016
2011	0.00	0.00	29,098
2012	0.00	0.00	104,421
2013	11.74	0.00	255,642
2014	0.00	0.00	543,023
2015	0.00	0.00	894,762
2016	0.00	0.00	1,755,993
2017	0.12	0.00	8,124,015
2018	0.50	0.00	40,395,501
2019	0.87	0.00	115,607,342
2020	2.26	0.00	190,065,703
2021	2.09	0.02	293,080,512
2022	2.24	0.03	274,129,622
2023^b^	3.16	0.02	152,536,208
**Grand Total**	**2.19**	**0.01**	**1,077,546,857**

Events are divided into adverse events and serious adverse events and reported based on the number of administrations in each container sold.

^a^ Number of administrations sold is assumed to be a close approximation to administrations consumed.

^b^ Usage data for 2023 is incomplete.

For interpretation of these results, it is important to note that regulatory restrictions on label statements affect the classification of dosage form animal health products in the NAERS™ system as the input classification is determined by the producer’s or retailer’s label. A product containing CBD may be labelled only as hemp or may include a qualifier such as broad-spectrum or full-spectrum, and not all products labelled as hemp contain CBD. NASC provides guidance to their membership that a broad-spectrum hemp extract contains “some or all of the compounds found naturally occurring in the plant, where THC has been processed to levels less than 0.3%” and a full-spectrum extract contains “all compounds found naturally occurring in the plant including, but not limited to, terpenes, cannabinoids and THC, where the cultivar’s THC level are grown or diluted to be less than 0.3%.” Administrations reported for broad-spectrum hemp products for dogs were 84,306,219 and for full-spectrum hemp products for dogs were 287,828,119. The hemp and hemp derivatives report is inclusive of AE for broad-spectrum and full spectrum hemp products, but the AE and SAE rates when calculated separately from the larger category are similar. Broad-spectrum hemp products had an AE and SAE rate of 2.40 and 0.02 per million administrations, respectively, and full-spectrum products had an AE and SAE rate of 2.83 and 0.03 per million administrations, respectively. Effectively, total administrations calculated for hemp and hemp-derivatives overestimates the post-market exposure of dogs to CBD products, and CBD total administrations underestimates the post-market exposure. Based on this information, it is reasonable to surmise that the rate of AE is between 1.6 and 2.8 per million administrations.

It is also important to separate animal health product AE from acute toxicosis due to marijuana (*Cannabis sativa* L. with a THC content higher than 0.3% by dried weight; defined in 21 CFR 1308.11) products intended for human consumption. Howard-Azzeh et al. analyzed factors influencing cannabis poisoning of dogs in the United States between 2009 and 2014 (4). The authors reported that an average of 1.12% of all calls to the Animal Poison Control Center were due to cannabis consumption and concluded that as cannabis products became more available for human consumption, the rate of poisoning in dogs increased.

The low AE rate reported in the NAERS™ system is supported by consumer survey data (1). Of respondents who had given their pet CBD, 45.3% indicated that they observed no side effects. The remaining side effect options included lethargy and sleepiness as the most common (24.2%, *n* = 116 each, participants could choose more than one answer). Other side effects were each indicated by <2% of respondents. In 2016, a similar survey of pet owners in the US via a link on a pet hemp product company website reported that 58.8% of survey respondents (*n* = 631) were currently using a hemp product for their dog. In this survey, pet owners reported sedation as the most common significant effect (53/278 respondents reporting sedation as “significant effect” vs. 4/278 reporting as “no effect,” however 190/278 reported this effect as “NA or do not know”). Although other side effects were reported, the authors reported that expense and ineffectiveness were the most common reasons for discontinuation of a product (36).

Variation in hemp product composition and quality could be responsible for differences in efficacy and safety. Botanical extracts prepared from hemp contain a number of phytochemicals including cannabinoids and terpenes, the levels of which can vary between extracts and can have a number of potential bioactivities (37). Extracts are susceptible to issues with product quality such as failure to follow good manufacturing practices, poor quality control, failure to screen for heavy metals, contamination from other plant products, etc. In a recent analysis of pet-specific cannabinoid products, CBD concentrations ranged from 0 to 66 mg/mL (including only oil delivery forms), which represented 0–154% of the label claim concentration. In addition, CBDA was found at high levels in two products (38). While quality control issues are outside the intent of this study, the lack of standardized products is a major hurdle for evaluating the safety of CBD products and supports the necessity of post-market surveillance.

Post market surveillance data and systems that provide continued vigilance are critical to monitor the risk of cannabinoid product use in animals. Even the most well defined and carefully conducted clinical studies cannot duplicate all possible scenarios or potential negative occurrences due to the use of products in the broader marketplace. The current study utilizes clinically relevant doses in a tolerability study to provide supportive baseline data for the evaluation of cannabinoids in domestic dogs. A limitation of this study is that only a single dose level is used for each product, although the dose level was chosen to be representative of real-world use of cannabinoid supplements in dogs. For clinicians and pet owners, information on the tolerability of different cannabinoids combinations can support informed use of these products. This study contributes to a data set demonstrating the safety of cannabinoids, which can be used to support future research in client-owned animals.

The results of the current study indicate that CBD, CBD + CBG and CBD + CBDA at the ratios and doses utilized were well tolerated when healthy male and female beagles were dosed for 90 consecutive days. These clinically determined conclusions are also supported by data from NAERS™ which is the most advanced system in the world for these types of products given to companion animals (specifically animals not intended for use in the human food chain).

Based on the data available it would be the conclusion of the authors that the substances do not pose significant risk to dogs in long-term use.

## Data availability statement

The original contributions presented in the study are included in the article/Supplementary material, further inquiries can be directed to the corresponding author.

## Ethics statement

The animal study was approved by the Institutional Animal Care and Use Committee of ClinVet USA LLC, an Association for Assessment and Accreditation of Laboratory Animal Care accredited facility. The study was conducted in accordance with the local legislation and institutional requirements.

## Author contributions

WB: Conceptualization, Funding acquisition, Resources, Writing – review & editing. MD: Methodology, Project administration, Supervision, Validation, Writing – original draft, Writing – review & editing. KV: Project administration, Writing – review & editing. JK-N: Writing – original draft.
