# Supplementary material for: Safety study of cannabidiol products in healthy dogs
Source: Front Vet Sci. 2024 Mar 1;11:1349590. doi: 10.3389/fvets.2024.1349590 (PMC10940325; doi:10.3389/fvets.2024.1349590)
Supplement: Supplementary file 1 [file Data_Sheet_1.docx]

**Supplementary Table 1 -** Summary of mean body weights (kg) for healthy beagles treated orally with MCT oil (Control; n=8) or 5 mg/kg bw/day of CBD (n=8), CBD + CBG (n=8) or CBD + CBDA (n=8) for 90 days, followed by 14 days without dosing.

| **Study day** | **Control** | **CBD** | **CBD + CBG** | **CBD + CBDA** |
| --- | --- | --- | --- | --- |
| -8 | 9.85 ± 1.098 | 9.65 ± 1.182 | 9.93 ± 1.321 | 10.04 ± 1.450 |
| -1 | 9.81 ± 1.108 | 9.73 ± 1.330 | 9.84 ± 1.320 | 10.03 ± 1.347 |
| 7 | 9.43 ± 1.123 | 9.43 ± 1.408 | 9.53 ± 1.223 | 9.73 ± 1.398 |
| 14 | 9.59 ± 1.152 | 9.61 ± 1.483 | 9.65 ± 1.283 | 10.04 ± 1.465 |
| 21 | 9.46 ± 1.177 | 9.75 ± 1.469 | 9.83 ± 1.266 | 10.04 ± 1.380 |
| 28 | 9.86 ± 1.221 | 9.55 ± 1.500 | 9.55 ± 1.329 | 9.96 ± 1.424 |
| 35 | 9.69 ± 1.292 | 9.71 ± 1.591 | 9.78 ± 1.346 | 10.46 ± 1.511 |
| 42 | 9.68 ± 1.296 | 9.61 ± 1.581 | 9.85 ± 1.330 | 10.11 ± 1.525 |
| 49 | 9.89 ± 1.361 | 9.73 ± 1.575 | 10.00 ± 1.394 | 10.29 ± 1.577 |
| 56 | 10.01 ± 1.474 | 9.85 ± 1.469 | 10.01 ± 1.398 | 10.40 ± 1.461 |
| 63 | 9.94 ± 1.407 | 10.01 ± 1.488 | 10.25 ± 1.435 | 10.59 ± 1.460 |
| 70 | 9.76 ± 1.311 | 9.64 ± 1.502 | 9.89 ± 1.385 | 10.16 ± 1.448 |
| 77 | 10.13 ± 1.349 | 9.95 ± 1.423 | 10.23 ± 1.525 | 10.46 ± 1.448 |
| 84 | 9.95 ± 1.330 | 9.59 ± 1.456 | 9.88 ± 1.511 | 10.03 ± 1.544 |
| 90 | 9.89 ± 1.296 | 9.59 ± 1.481 | 9.88 ± 1.421 | 10.09 ± 1.502 |
| 97 | 10.15 ± 1.319 | 10.09 ± 1.371 | 10.36 ± 1.380 | 10.63 ± 1.460 |
| 104 | 10.23 ± 1.351 | 10.04 ± 1.245 | 10.38 ± 1.370 | 10.54 ± 1.538 |

Data represents body weights in kg. Data are reported as mean ± SD.

CBD = Cannabidiol. CBDA = Cannabidiolic acid. CBG = Cannabigerol. MCT = Medium chain triglyceride.

**Supplementary Table 2 -** Summary of mean weekly average food consumption (g) for healthy beagles treated orally with MCT oil (Control; n=8) or 5 mg/kg bw/day of CBD (n=8), CBD + CBG (n=8) or CBD + CBDA (n=8) for 90 days, followed by 14 days without dosing.

| **Study week** | **Control** | **CBD** | **CBD + CBG** | **CBD + CBDA** |
| --- | --- | --- | --- | --- |
| Acclimatization (Days -7 to -1) | 72.385 ± 35.034 | 95.719 ± 53.902 | 56.929 ± 53.548 | 111.519 ± 89.402 |
| 1  (Days 0 to 6) | 249.213 ± 107.876 | 149.491 ± 27.775 | 188.243 ± 77.973 | 168.248 ± 46.885 |
| 2  (Days 7 to 13) | 235.982 ± 78.288 | 171.833 ± 22.414 | 194.166 ± 76.649 | 212.766 ± 44.140 |
| 3  (Days 14 to 20) | 235.789 ± 73.419 | 176.264 ± 29.678 | 196.057 ± 80.280 | 211.308 ± 32.626 |
| 4  (Days 21 to 27) | 243.382 ± 103.380 | 196.852 ± 36.748 | 205.622 ± 70.953 | 225.554 ± 41.031 |
| 5  (Days 28 to 34) | 230.093 ± 101.275 | 169.803 ± 37.325 | 201.159 ± 61.653 | 202.213 ± 42.499 |
| 6  (Days 35 to 41) | 256.759 ± 90.778 | 181.954 ± 28.804 | 202.850 ± 41.052 | 213.471 ± 51.121 |
| 7  (Days 42 to 48) | 240.908 ± 90.122 | 171.325 ± 16.430 | 197.524 ± 41.568 | 202.639 ± 53.673 |
| 8  (Days 49 to 55) | 243.482 ± 103.172 | 184.693 ± 13.231 | 204.345 ± 53.126 | 238.258 ± 48.260 |
| 9  (Days 56 to 62) | 251.690 ± 91.825 | 182.386 ± 21.868 | 213.913 ± 53.126 | 210.239 ± 54.388 |
| 10  (Days 63 to 69) | 253.394 ± 92.140 | 152.425 ± 20.016 | 192.845 ± 55.010 | 194.400 ± 54.266 |
| 11  (Days 70 to 76) | 252.908 ± 68.652 | 172.680 ± 35.051 | 193.882 ± 51.587 | 191.234 ± 40.786 |
| 12  (Days 77 to 83) | 230.437 ± 95.399 | 165.521 ± 12.561 | 180.040 ± 38.502 | 196.384 ± 43.078 |
| 13  (Days 84 to 90) | 236.176 ± 79.456 | 172.888 ± 17.790 | 215.811 ± 56.459 | 207.738 ± 49.110 |

Food consumption was determined by weighing food prior to and after feeding each animal daily from day -7 through the end of the study. Data represents weekly average food consumption in g. Data are reported as mean ± SD.

CBD = Cannabidiol. CBDA = Cannabidiolic acid. CBG = Cannabigerol. MCT = Medium chain triglyceride.

**Supplementary Table 3 -** Mean hematology results for healthy beagles treated orally with MCT oil (Control; n=8) or 5 mg/kg bw/day of CBD (n=8), CBD + CBG (n=8) or CBD + CBDA (n=8) for 90 days, followed by 14 days without dosing. Baseline is study day -9 for the purposes of statistical comparison.

| **Study day** | **Control** | **CBD** | **CBD + CBG** | **CBD + CBDA** |
| --- | --- | --- | --- | --- |
| **Basophils (thousand/µL)** | | | | |
| -9 | 0.063 ± 0.052 | 0.075 ± 0.046 | 0.050 ± 0.053 | 0.063 ± 0.052 |
| 14 | 0.000 ± 0.000* | 0.025 ± 0.046* | 0.029 ± 0.049 | 0.029 ± 0.049 |
| 28 | 0.000 ± 0.000* | 0.025 ± 0.046* | 0.000 ± 0.000* | 0.000 ± 0.000* |
| 56 | 0.029 ± 0.049 | 0.043 ± 0.053 | 0.025 ± 0.046 | 0.050 ± 0.076 |
| 90 | 0.025 ± 0.046 | 0.050 ± 0.053 | 0.013 ± 0.035* | 0.050 ± 0.053 |
| 104 | 0.025 ± 0.046 | 0.075 ± 0.046 | 0.063 ± 0.052 | 0.088 ± 0.035 |
| **Eosinophils (thousand/µL)** | | | | |
| -9 | 0.250 ± 0.107 | 0.300 ± 0.227 | 0.350 ± 0.169 | 0.263 ± 0.141 |
| 14 | 0.225 ± 0.089 | 0.325 ± 0.128 | 0.286 ± 0.069 | 0.371 ± 0.138 |
| 28 | 0.238 ± 0.074 | 0.363 ± 0.151 | 0.263 ± 0.092 | 0.388 ± 0.217 |
| 56 | 0.300 ± 0.153 | 0.429 ± 0.160 | 0.325 ± 0.089 | 0.463 ± 0.256* |
| 90 | 0.350 ± 0.185 | 0.438 ± 0.192 | 0.313 ± 0.083 | 0.425 ± 0.139* |
| 104 | 0.363 ± 0.239* | 0.450 ± 0.141 | 0.413 ± 0.146 | 0.463 ± 0.141* |
| **Hct (%)** | | | | |
| -9 | 48.1 ± 2.90 | 50.6 ± 2.72 | 50.1 ± 3.87 | 49.8 ± 2.82 |
| 14 | 49.8 ± 2.60 | 50.8 ± 3.15 | 48.7 ± 3.95 | 47.4 ± 2.51 |
| 28 | 53.0 ± 5.42* | 53.9 ± 2.30* | 52.5 ± 4.28* | 52.5 ± 4.87 |
| 56 | 51.1 ± 6.18* | 49.6 ± 3.51 | 49.3 ± 3.81 | 50.1 ± 4.36 |
| 90 | 54.0 ± 3.21* | 52.0 ± 3.85 | 51.9 ± 3.52 | 52.1 ± 3.87 |
| 104 | 53.5 ± 3.78* | 52.6 ± 4.47 | 51.1 ± 3.91 | 51.5 ± 3.21 |
| **Hemoglobin (g/dL)** | | | | |
| -9 | 16.188 ± 0.906 | 16.900 ± 0.964 | 16.763 ± 1.260 | 16.675 ± 1.040 |
| 14 | 16.150 ± 1.073 | 16.275 ± 1.163 | 16.014 ± 1.067* | 15.643 ± 0.954* |
| 28 | 17.188 ± 1.847* | 17.375 ± 0.965 | 16.850 ± 1.295 | 16.838 ± 1.798 |
| 56 | 17.029 ± 2.070 | 16.614 ± 1.208 | 16.463 ± 1.240 | 16.575 ± 1.537 |
| 90 | 17.613 ± 1.023* | 16.775 ± 1.079 | 16.500 ± 0.958 | 16.750 ± 1.322 |
| 104 | 17.288 ± 1.233* | 16.788 ± 1.243 | 16.350 ± 1.206 | 16.288 ± 0.970 |
| **Large unstained cells (thousand/µL)** | | | | |
| -9 | 0.013 ± 0.035 | 0.000 ± 0.000 | 0.025 ± 0.046 | 0.000 ± 0.000 |
| 14 | 0.013 ± 0.035 | 0.025 ± 0.046 | 0.014 ± 0.038 | 0.014 ± 0.038 |
| 28 | 0.000 ± 0.000 | 0.038 ± 0.052* | 0.013 ± 0.035 | 0.025 ± 0.046 |
| 56 | 0.000 ± 0.000 | 0.000 ± 0.000 | 0.000 ± 0.000 | 0.025 ± 0.046 |
| 90 | 0.000 ± 0.000 | 0.013 ± 0.035 | 0.000 ± 0.000 | 0.013 ± 0.035 |
| 104 | 0.000 ± 0.000 | 0.013 ± 0.035 | 0.000 ± 0.000 | 0.025 ± 0.046 |
| **Lymphocytes (thousand/µL)** | | | | |
| -9 | 2.050 ± 0.701 | 2.513 ± 0.844 | 2.025 ± 0.406 | 2.188 ± 0.591 |
| 14 | 1.938 ± 0.472 | 2.575 ± 0.658 | 2.143 ± 0.424 | 2.329 ± 0.304 |
| 28 | 2.075 ± 0.492 | 2.763 ± 0.713 | 2.175 ± 0.362 | 2.113 ± 0.364 |
| 56 | 2.000 ± 0.520 | 2.729 ± 0.778 | 2.150 ± 0.396 | 2.338 ± 0.358 |
| 90 | 1.963 ± 0.717 | 2.725 ± 0.808 | 2.225 ± 0.489 | 2.300 ± 0.590 |
| 104 | 2.500 ± 0.760* | 3.025 ± 1.028* | 2.388 ± 0.376* | 2.413 ± 0.432 |
| **Mean cell hemoglobin (pg)** | | | | |
| -9 | 24.3 ± 0.46 | 24.6 ± 0.92 | 24.1 ± 0.64 | 24.5 ± 0.93 |
| 14 | 24.1 ± 0.35 | 24.3 ± 0.71* | 24.1 ± 0.38 | 24.4 ± 0.98 |
| 28 | 24.1 ± 0.35 | 24.3 ± 0.71* | 24.0 ± 0.53 | 24.4 ± 0.92 |
| 56 | 23.9 ± 0.69* | 24.6 ± 0.53 | 24.1 ± 0.64 | 24.3 ± 0.71 |
| 90 | 23.9 ± 0.64* | 24.4 ± 0.52 | 23.9 ± 0.64 | 23.9 ± 0.64* |
| 104 | 23.8 ± 0.71* | 24.3 ± 0.46* | 24.1 ± 0.64 | 24.1 ± 0.64* |
| **Mean cell hemoglobin concentration (g/dL)** | | | | |
| -9 | 33.6 ± 0.52 | 33.5 ± 0.53 | 33.5 ± 0.53 | 33.5 ± 0.53 |
| 14 | 32.4 ± 0.52* | 32.3 ± 0.71* | 33.0 ± 1.00 | 33.1 ± 0.69 |
| 28 | 32.3 ± 0.46* | 32.1 ± 0.83* | 32.1 ± 0.35* | 32.1 ± 0.35* |
| 56 | 33.4 ± 0.53 | 33.3 ± 0.49 | 33.4 ± 0.52 | 33.1 ± 0.64 |
| 90 | 32.6 ± 0.52* | 32.4 ± 0.52* | 31.9 ± 0.83* | 32.3 ± 0.46* |
| 104 | 32.3 ± 0.46* | 31.8 ± 0.46* | 32.0 ± 0.00* | 31.9 ± 0.35* |
| **Mean cell volume (fL)** | | | | |
| -9 | 72.1 ± 0.99 | 73.8 ± 1.67 | 72.3 ± 1.67 | 73.5 ± 2.20 |
| 14 | 74.8 ± 1.49* | 75.6 ± 1.51* | 73.1 ± 1.07 | 74.0 ± 2.24 |
| 28 | 74.3 ± 1.67* | 76.1 ± 2.53* | 75.3 ± 1.49* | 75.8 ± 2.71* |
| 56 | 72.0 ± 2.08 | 73.1 ± 1.95 | 72.9 ± 1.64 | 73.1 ± 1.81 |
| 90 | 73.3 ± 1.98* | 75.3 ± 1.98* | 74.6 ± 1.77* | 75.3 ± 1.83* |
| 104 | 73.9 ± 2.03* | 75.8 ± 1.91* | 74.9 ± 1.73* | 75.5 ± 2.27* |
| **Mean platelet volume (fL)** | | | | |
| -9 | 10.088 ± 1.152 | 9.575 ± 0.568 | 9.650 ± 0.859 | 9.675 ± 0.709 |
| 14 | 12.250 ± 1.146* | 10.788 ± 0.955* | 10.471 ± 1.174* | 10.557 ± 1.050* |
| 28 | 12.425 ± 1.208* | 11.888 ± 0.653* | 12.000 ± 0.878* | 12.538 ± 0.802* |
| 56 | 11.800 ± 2.235* | 9.743 ± 0.547 | 10.013 ± 0.834 | 10.050 ± 0.786 |
| 90 | 11.863 ± 1.452* | 11.000 ± 1.193* | 11.913 ± 1.239* | 12.088 ± 1.480* |
| 104 | 11.013 ± 0.955* | 10.838 ± 0.674* | 10.988 ± 0.785* | 11.275 ± 0.595* |
| **Monocytes (thousand/µL)** | | | | |
| -9 | 0.450 ± 0.093 | 0.550 ± 0.214 | 0.400 ± 0.107 | 0.488 ± 0.099 |
| 14 | 0.313 ± 0.083* | 0.513 ± 0.113 | 0.343 ± 0.127 | 0.429 ± 0.160* |
| 28 | 0.325 ± 0.104* | 0.425 ± 0.089 | 0.363 ± 0.141 | 0.400 ± 0.107 |
| 56 | 0.329 ± 0.111* | 0.457 ± 0.215 | 0.350 ± 0.093 | 0.463 ± 0.151 |
| 90 | 0.400 ± 0.251 | 0.525 ± 0.175 | 0.388 ± 0.099 | 0.400 ± 0.151* |
| 104 | 0.388 ± 0.125 | 0.600 ± 0.351 | 0.438 ± 0.220 | 0.375 ± 0.104* |
| **Platelet count (thousand/µL)** | | | | |
| -9 | 303.8 ± 81.67 | 374.3 ± 75.14 | 319.8 ± 44.76 | 325.1 ± 81.36 |
| 14 | 294.5 ± 55.07 | 343.3 ± 80.05 | 318.6 ± 64.59 | 317.7 ± 50.86 |
| 28 | 299.3 ± 42.29 | 361.1 ± 80.32 | 326.6 ± 58.75 | 311.8 ± 65.40 |
| 56 | 312.1 ± 54.47 | 378.1 ± 72.49 | 329.5 ± 54.98 | 328.6 ± 46.53 |
| 90 | 269.5 ± 52.30 | 369.0 ± 77.27 | 304.4 ± 43.00 | 327.5 ± 96.92 |
| 104 | 291.3 ± 36.00 | 340.6 ± 65.00* | 322.3 ± 26.35 | 333.4 ± 95.31 |
| **RBC count (million/µL)** | | | | |
| -9 | 6.688 ± 0.412 | 6.863 ± 0.338 | 6.938 ± 0.507 | 6.825 ± 0.523 |
| 14 | 6.663 ± 0.424 | 6.700 ± 0.366 | 6.657 ± 0.500* | 6.414 ± 0.406* |
| 28 | 7.150 ± 0.860* | 7.088 ± 0.398 | 7.000 ± 0.626 | 6.938 ± 0.833 |
| 56 | 7.114 ± 1.017* | 6.800 ± 0.365 | 6.788 ± 0.541 | 6.838 ± 0.652 |
| 90 | 7.338 ± 0.501* | 6.925 ± 0.369 | 6.950 ± 0.460 | 6.950 ± 0.586 |
| 104 | 7.263 ± 0.576* | 6.950 ± 0.472 | 6.838 ± 0.553 | 6.813 ± 0.514 |
| **Red cell distribution width (%)** | | | | |
| -9 | 12.788 ± 0.290 | 12.688 ± 0.458 | 12.563 ± 0.498 | 12.438 ± 0.441 |
| 14 | 12.013 ± 0.275* | 12.050 ± 0.374* | 12.043 ± 0.199* | 12.086 ± 0.339 |
| 28 | 12.113 ± 0.264* | 11.975 ± 0.582* | 12.075 ± 0.292* | 12.150 ± 0.621 |
| 56 | 12.129 ± 0.407* | 12.229 ± 0.791* | 12.138 ± 0.478* | 12.238 ± 0.478 |
| 90 | 12.338 ± 0.396* | 11.975 ± 0.362* | 12.038 ± 0.407* | 12.088 ± 0.309 |
| 104 | 12.575 ± 0.349 | 12.338 ± 0.450 | 12.350 ± 0.487 | 12.400 ± 0.131 |
| **Segmented neutrophils (thousand/µL)** | | | | |
| -9 | 7.013 ± 1.315 | 7.363 ± 2.927 | 6.300 ± 2.287 | 7.138 ± 1.880 |
| 14 | 4.513 ± 0.511* | 5.375 ± 1.305 | 4.643 ± 1.222* | 6.271 ± 1.687 |
| 28 | 4.550 ± 1.027* | 6.288 ± 1.740 | 5.088 ± 1.715* | 6.163 ± 1.716 |
| 56 | 4.743 ± 0.772* | 6.300 ± 2.416 | 5.163 ± 1.333* | 6.950 ± 1.724 |
| 90 | 6.163 ± 1.717 | 6.863 ± 1.650 | 5.363 ± 1.296* | 7.213 ± 2.942 |
| 104 | 6.075 ± 1.368 | 7.400 ± 4.498 | 6.125 ± 2.408 | 6.813 ± 2.013 |
| **WBC count (thousand/µL)** | | | | |
| -9 | 9.875 ± 1.908 | 10.775 ± 3.986 | 9.125 ± 2.505 | 10.163 ± 2.224 |
| 14 | 7.038 ± 0.450* | 8.875 ± 2.091 | 7.443 ± 1.422* | 9.471 ± 1.911 |
| 28 | 7.250 ± 1.181* | 9.950 ± 2.396 | 7.938 ± 1.973* | 9.138 ± 1.968 |
| 56 | 7.443 ± 1.243* | 10.043 ± 3.201 | 8.075 ± 1.411 | 10.288 ± 2.157 |
| 90 | 8.925 ± 2.183 | 10.638 ± 2.244 | 8.350 ± 1.422 | 10.413 ± 3.634 |
| 104 | 9.400 ± 2.019 | 11.588 ± 5.195 | 9.438 ± 2.894 | 10.175 ± 2.449 |

Data are reported as mean ± SD of hematology results on each assessment day.

*Significantly different from baseline (P < .05).

µL = microliter. CBD = Cannabidiol. CBDA = Cannabidiolic acid. CBG = Cannabigerol. dL = Deciliter. fL = Femtoliter. g = gram. MCT = Medium chain triglyceride. Pg = picogram.

**Supplementary Table 4** **-** Mean clinical chemistry results for healthy beagles treated orally with MCT oil (Control; n=8) or 5 mg/kg bw/day of CBD (n=8), CBD + CBG (n=8) or CBD + CBDA (n=8) for 90 days, followed by 14 days without dosing. Baseline is study day -9 for the purposes of statistical comparison.

| **Study day** | **Control** | **CBD** | **CBD + CBG** | **CBD + CBDA** |
| --- | --- | --- | --- | --- |
| **Albumin/globulin ratio** | | | | |
| -9 | 2.050 ± 0.273 | 2.000 ± 0.302 | 2.088 ± 0.173 | 1.925 ± 0.183 |
| 14 | 2.175 ± 0.255 | 2.263 ± 0.400* | 2.150 ± 0.151 | 1.963 ± 0.213 |
| 28 | 2.100 ± 0.200 | 2.025 ± 0.406 | 2.138 ± 0.213 | 1.988 ± 0.196 |
| 56 | 2.238 ± 0.239* | 1.913 ± 0.259 | 1.988 ± 0.217 | 1.775 ± 0.198 |
| 90 | 1.900 ± 0.256* | 1.688 ± 0.394* | 1.788 ± 0.155* | 1.688 ± 0.181* |
| 104 | 1.938 ± 0.226 | 1.838 ± 0.358 | 1.850 ± 0.239* | 1.838 ± 0.316 |
| **ALT (U/L)** | | | | |
| -9 | 33.3 ± 9.74 | 39.8 ± 13.76 | 42.4 ± 12.76 | 40.6 ± 15.10 |
| 14 | 30.1 ± 8.44 | 36.3 ± 15.12 | 33.5 ± 7.03* | 31.9 ± 10.51* |
| 28 | 34.6 ± 8.70 | 35.3 ± 11.26 | 35.4 ± 7.96* | 36.8 ± 14.79* |
| 56 | 33.9 ± 9.01 | 34.6 ± 8.62 | 35.1 ± 7.36* | 34.4 ± 11.98* |
| 90 | 37.3 ± 10.15 | 36.1 ± 11.42 | 39.5 ± 8.47 | 35.6 ± 11.03* |
| 104 | 36.5 ± 9.23 | 42.0 ± 13.89 | 35.3 ± 6.36* | 38.0 ± 12.20 |
| **AST (U/L)** | | | | |
| -9 | 28.0 ± 7.29 | 29.3 ± 6.61 | 27.0 ± 6.28 | 29.8 ± 5.42 |
| 14 | 33.1 ± 5.46* | 34.5 ± 10.89 | 29.0 ± 3.02 | 35.3 ± 12.02* |
| 28 | 30.1 ± 3.68 | 30.1 ± 6.45 | 29.4 ± 2.67 | 33.4 ± 7.58 |
| 56 | 30.4 ± 2.88 | 30.6 ± 6.16 | 29.0 ± 2.73 | 30.6 ± 5.80 |
| 90 | 29.5 ± 3.12 | 28.0 ± 3.74 | 30.3 ± 3.11 | 31.8 ± 4.06 |
| 104 | 33.3 ± 5.68* | 35.8 ± 9.27 | 36.0 ± 5.24* | 33.5 ± 5.15 |
| **Albumin (g/dL)** | | | | |
| -9 | 3.925 ± 0.089 | 3.838 ± 0.200 | 3.950 ± 0.169 | 3.813 ± 0.146 |
| 14 | 3.888 ± 0.125 | 3.800 ± 0.214 | 3.875 ± 0.191 | 3.588 ± 0.125* |
| 28 | 3.950 ± 0.207 | 3.725 ± 0.231 | 3.913 ± 0.223 | 3.700 ± 0.120 |
| 56 | 3.975 ± 0.128 | 3.725 ± 0.198 | 3.825 ± 0.205 | 3.613 ± 0.125* |
| 90 | 3.863 ± 0.130 | 3.550 ± 0.302* | 3.750 ± 0.256* | 3.575 ± 0.219* |
| 104 | 3.913 ± 0.113 | 3.713 ± 0.203 | 3.713 ± 0.331* | 3.638 ± 0.169* |
| **ALP (U/L)** | | | | |
| -9 | 23.9 ± 10.60 | 31.1 ± 10.51 | 28.8 ± 9.60 | 31.1 ± 7.04 |
| 14 | 21.0 ± 8.98 | 52.6 ± 46.72 | 29.0 ± 8.42 | 53.1 ± 49.74* |
| 28 | 22.5 ± 10.07 | 87.6 ± 94.67* | 33.5 ± 11.50 | 62.8 ± 42.03* |
| 56 | 21.6 ± 8.28 | 72.9 ± 64.36* | 34.9 ± 13.48 | 65.9 ± 29.53* |
| 90 | 23.3 ± 10.82 | 69.6 ± 57.72* | 34.6 ± 11.58 | 50.8 ± 27.31 |
| 104 | 25.1 ± 12.61 | 42.0 ± 20.91 | 38.4 ± 27.41* | 37.8 ± 15.23 |
| **Amylase (U/L)** | | | | |
| -9 | 612.5 ± 216.34 | 509.5 ± 81.08 | 499.8 ± 97.63 | 592.5 ± 113.46 |
| 14 | 612.1 ± 209.83 | 534.4 ± 77.07 | 513.8 ± 110.57 | 584.5 ± 79.79 |
| 28 | 658.0 ± 264.21 | 559.0 ± 129.59 | 547.1 ± 109.59 | 606.5 ± 84.69 |
| 56 | 682.9 ± 299.70 | 530.6 ± 83.32 | 549.1 ± 139.95 | 636.0 ± 80.11 |
| 90 | 711.0 ± 217.78* | 580.3 ± 116.22* | 547.6 ± 84.83 | 653.4 ± 112.89 |
| 104 | 687.3 ± 260.29* | 539.5 ± 104.77 | 530.1 ± 119.82 | 585.0 ± 73.85 |
| **Anion gap (mEq/L)** | | | | |
| -9 | 19.4 ± 1.06 | 19.5 ± 1.20 | 19.6 ± 1.19 | 19.1 ± 0.83 |
| 14 | 5.4 ± 4.07* | 9.8 ± 5.42* | 5.4 ± 0.92* | 6.5 ± 1.85* |
| 28 | 18.6 ± 1.30 | 19.3 ± 2.12 | 20.0 ± 1.31 | 19.0 ± 1.41 |
| 56 | 21.1 ± 1.13 | 21.0 ± 1.51 | 20.9 ± 1.13 | 20.1 ± 1.13 |
| 90 | 21.4 ± 1.69 | 19.9 ± 1.25 | 20.9 ± 1.55 | 20.3 ± 2.31 |
| 104 | 14.3 ± 3.81* | 14.4 ± 3.93* | 14.5 ± 2.20* | 16.8 ± 2.19* |
| **Bicarbonate (mEq/L)** | | | | |
| -9 | 19.6 ± 1.77 | 20.5 ± 1.31 | 19.9 ± 1.36 | 20.1 ± 1.55 |
| 14 | 30.3 ± 3.24* | 27.8 ± 4.65* | 30.6 ± 1.77* | 29.0 ± 2.39* |
| 28 | 20.9 ± 1.13 | 21.3 ± 1.91 | 19.9 ± 1.46 | 20.3 ± 1.04 |
| 56 | 18.9 ± 1.73 | 20.3 ± 1.75 | 19.0 ± 0.93 | 19.4 ± 0.52 |
| 90 | 18.8 ± 1.49 | 20.9 ± 1.25 | 19.3 ± 0.89 | 20.5 ± 0.93 |
| 104 | 24.0 ± 2.93* | 24.5 ± 3.38* | 23.6 ± 2.39* | 21.8 ± 2.05* |
| **Calcium (mg/dL)** | | | | |
| -9 | 10.638 ± 0.233 | 10.400 ± 0.262 | 10.525 ± 0.212 | 10.463 ± 0.316 |
| 14 | 10.488 ± 0.327 | 10.175 ± 0.328* | 10.375 ± 0.191 | 10.150 ± 0.278* |
| 28 | 10.675 ± 0.311 | 10.225 ± 0.238 | 10.425 ± 0.149 | 10.325 ± 0.225 |
| 56 | 10.488 ± 0.314 | 10.375 ± 0.306 | 10.463 ± 0.220 | 10.388 ± 0.331 |
| 90 | 10.463 ± 0.329 | 10.200 ± 0.273* | 10.400 ± 0.262 | 10.388 ± 0.253 |
| 104 | 10.575 ± 0.369 | 10.413 ± 0.164 | 10.325 ± 0.410 | 10.338 ± 0.200 |
| **Chloride (mEq/L)** | | | | |
| -9 | 111.9 ± 1.13 | 111.1 ± 0.83 | 111.8 ± 1.67 | 111.5 ± 2.00 |
| 14 | 115.0 ± 2.07* | 113.4 ± 2.26* | 115.0 ± 1.77* | 115.3 ± 1.91* |
| 28 | 111.6 ± 2.13 | 110.3 ± 0.89 | 111.4 ± 1.51 | 111.9 ± 1.13 |
| 56 | 110.8 ± 1.58 | 110.6 ± 1.06 | 112.1 ± 1.46 | 112.6 ± 2.00 |
| 90 | 112.5 ± 1.60 | 112.8 ± 2.25 | 114.4 ± 1.77* | 113.8 ± 2.55* |
| 104 | 113.5 ± 2.45 | 112.0 ± 2.20 | 112.9 ± 1.81 | 112.6 ± 1.19 |
| **Cholesterol (mg/dL)** | | | | |
| -9 | 185.3 ± 49.56 | 169.5 ± 37.94 | 174.6 ± 36.47 | 161.5 ± 30.21 |
| 14 | 181.3 ± 66.34 | 180.3 ± 63.65 | 174.9 ± 63.12 | 157.9 ± 25.07 |
| 28 | 190.0 ± 58.73 | 190.3 ± 64.62 | 191.9 ± 71.36 | 173.8 ± 26.21 |
| 56 | 173.4 ± 46.85 | 180.1 ± 59.68 | 181.6 ± 58.56 | 168.0 ± 27.49 |
| 90 | 167.1 ± 38.81 | 182.6 ± 46.22 | 170.1 ± 47.44 | 157.3 ± 22.00 |
| 104 | 177.1 ± 49.85 | 190.5 ± 57.95 | 175.9 ± 61.06 | 159.8 ± 52.89 |
| **Creatine kinase (U/L)** | | | | |
| -9 | 270.9 ± 137.85 | 284.3 ± 162.92 | 216.6 ± 152.83 | 261.4 ± 145.50 |
| 14 | 425.5 ± 268.05* | 518.5 ± 519.01 | 231.8 ± 59.94 | 455.4 ± 375.88* |
| 28 | 252.8 ± 114.91 | 280.3 ± 158.65 | 211.9 ± 59.95 | 242.8 ± 92.45 |
| 56 | 222.0 ± 62.92 | 226.5 ± 84.54 | 183.0 ± 46.43 | 228.9 ± 87.04 |
| 90 | 183.8 ± 44.17 | 174.4 ± 36.28 | 152.3 ± 42.55 | 197.9 ± 54.36 |
| 104 | 278.0 ± 120.94 | 366.3 ± 175.96 | 385.3 ± 226.80* | 256.5 ± 46.22 |
| **Creatinine (mg/dL)** | | | | |
| -9 | 0.613 ± 0.064 | 0.613 ± 0.064 | 0.600 ± 0.093 | 0.638 ± 0.052 |
| 14 | 0.638 ± 0.074 | 0.588 ± 0.083 | 0.600 ± 0.076 | 0.613 ± 0.083 |
| 28 | 0.650 ± 0.093 | 0.600 ± 0.076 | 0.613 ± 0.064 | 0.675 ± 0.046 |
| 56 | 0.663 ± 0.106* | 0.625 ± 0.089 | 0.625 ± 0.089 | 0.675 ± 0.071 |
| 90 | 0.688 ± 0.064* | 0.638 ± 0.119 | 0.650 ± 0.076* | 0.700 ± 0.076* |
| 104 | 0.663 ± 0.052* | 0.600 ± 0.076 | 0.588 ± 0.083 | 0.650 ± 0.093 |
| **Direct bilirubin (mg/dL)** | | | | |
| -9 | 0.050 ± 0.053 | 0.038 ± 0.052 | 0.013 ± 0.035 | 0.025 ± 0.046 |
| 14 | 0.000 ± 0.000* | 0.000 ± 0.000* | 0.000 ± 0.000 | 0.000 ± 0.000* |
| 28 | 0.000 ± 0.000* | 0.000 ± 0.000* | 0.000 ± 0.000 | 0.000 ± 0.000* |
| 56 | 0.000 ± 0.000* | 0.000 ± 0.000* | 0.000 ± 0.000 | 0.000 ± 0.000* |
| 90 | 0.000 ± 0.000* | 0.000 ± 0.000* | 0.000 ± 0.000 | 0.000 ± 0.000* |
| 104 | 0.000 ± 0.000* | 0.000 ± 0.000* | 0.000 ± 0.000 | 0.000 ± 0.000* |
| **GGT (U/L)** | | | | |
| -9 | 2.5 ± 1.31 | 2.0 ± 0.93 | 2.5 ± 0.93 | 2.4 ± 1.41 |
| 14 | 2.0 ± 0.53 | 2.1 ± 1.25 | 2.9 ± 0.83 | 2.1 ± 1.13 |
| 28 | 0.8 ± 0.71* | 1.9 ± 0.64 | 1.9 ± 0.64 | 1.8 ± 0.89 |
| 56 | 2.5 ± 1.60 | 2.9 ± 1.25 | 2.9 ± 0.83 | 2.6 ± 0.52 |
| 90 | 2.8 ± 0.89 | 2.9 ± 0.83 | 2.9 ± 0.35 | 2.6 ± 1.69 |
| 104 | 2.6 ± 0.74 | 2.6 ± 0.92 | 1.8 ± 0.89* | 1.8 ± 1.04 |
| **Globulin (g/dL)** | | | | |
| -9 | 1.950 ± 0.239 | 1.963 ± 0.239 | 1.925 ± 0.175 | 2.000 ± 0.185 |
| 14 | 1.825 ± 0.225* | 1.713 ± 0.247* | 1.813 ± 0.125 | 1.850 ± 0.169* |
| 28 | 1.900 ± 0.214 | 1.925 ± 0.373 | 1.850 ± 0.169 | 1.875 ± 0.175 |
| 56 | 1.800 ± 0.239* | 1.975 ± 0.287 | 1.938 ± 0.160 | 2.050 ± 0.207 |
| 90 | 2.075 ± 0.255* | 2.175 ± 0.354* | 2.113 ± 0.099* | 2.138 ± 0.226 |
| 104 | 2.038 ± 0.256 | 2.075 ± 0.377 | 2.038 ± 0.213 | 2.038 ± 0.354 |
| **Glucose (mg/dL)** | | | | |
| -9 | 89.3 ± 9.44 | 87.5 ± 8.05 | 90.6 ± 5.42 | 93.3 ± 11.06 |
| 14 | 83.1 ± 5.28* | 77.8 ± 9.00* | 83.4 ± 6.05* | 85.8 ± 4.10* |
| 28 | 81.6 ± 9.84* | 80.9 ± 7.57* | 80.8 ± 7.67* | 87.6 ± 3.85 |
| 56 | 79.6 ± 8.28* | 78.1 ± 8.29* | 85.4 ± 5.90* | 87.5 ± 5.66* |
| 90 | 79.4 ± 7.93* | 79.1 ± 6.27* | 85.0 ± 7.71* | 86.5 ± 5.76* |
| 104 | 78.9 ± 7.20* | 70.4 ± 9.41* | 79.9 ± 3.68* | 82.4 ± 6.59* |
| **Indirect bilirubin (mg/dL)** | | | | |
| -9 | 0.013 ± 0.035 | 0.000 ± 0.000 | 0.025 ± 0.046 | 0.000 ± 0.000 |
| 14 | 0.075 ± 0.046* | 0.025 ± 0.046 | 0.100 ± 0.000 | 0.025 ± 0.046 |
| 28 | 0.050 ± 0.053 | 0.063 ± 0.052* | 0.063 ± 0.052 | 0.050 ± 0.053* |
| 56 | 0.063 ± 0.052* | 0.000 ± 0.000 | 0.163 ± 0.342 | 0.000 ± 0.000 |
| 90 | 0.025 ± 0.046 | 0.000 ± 0.000 | 0.000 ± 0.000 | 0.013 ± 0.035 |
| 104 | 0.038 ± 0.052 | 0.050 ± 0.053* | 0.038 ± 0.052 | 0.025 ± 0.046 |
| **Iron (µg/dL)** | | | | |
| -9 | 139.4 ± 21.78 | 153.5 ± 34.62 | 152.3 ± 20.55 | 144.9 ± 33.79 |
| 14 | 205.4 ± 51.44* | 211.8 ± 48.33* | 191.4 ± 42.80 | 172.3 ± 36.29* |
| 28 | 214.5 ± 54.85* | 216.9 ± 36.26* | 211.9 ± 53.83* | 159.1 ± 36.00 |
| 56 | 197.8 ± 23.35* | 198.4 ± 50.47* | 189.0 ± 35.90 | 183.5 ± 40.73* |
| 90 | 170.4 ± 51.52 | 229.1 ± 55.77* | 199.3 ± 33.96* | 165.9 ± 31.43 |
| 104 | 187.4 ± 36.00* | 192.6 ± 57.51 | 203.4 ± 63.14* | 173.1 ± 28.96* |
| **LDH (U/L)** | | | | |
| -9 | 151.6 ± 81.94 | 180.8 ± 54.75 | 107.9 ± 39.28 | 137.6 ± 43.01 |
| 14 | 259.1 ± 133.25* | 250.3 ± 105.43 | 150.6 ± 59.92* | 183.8 ± 78.60 |
| 28 | 203.5 ± 121.78 | 210.3 ± 98.49 | 132.8 ± 42.94 | 151.5 ± 54.56 |
| 56 | 177.4 ± 53.84 | 214.9 ± 144.70 | 103.9 ± 42.00 | 124.4 ± 46.21 |
| 90 | 142.1 ± 20.27 | 166.6 ± 63.36 | 94.4 ± 31.79 | 165.3 ± 66.22 |
| 104 | 225.5 ± 88.51* | 257.9 ± 102.07* | 162.3 ± 65.56* | 185.5 ± 76.70 |
| **Lipase (U/L)** | | | | |
| -9 | 61.1 ± 42.29 | 40.3 ± 21.54 | 32.6 ± 9.07 | 48.1 ± 21.56 |
| 14 | 51.0 ± 31.25 | 45.9 ± 33.07 | 36.3 ± 13.49 | 41.8 ± 17.33 |
| 28 | 54.4 ± 35.50 | 71.0 ± 82.00 | 49.1 ± 24.69* | 49.1 ± 20.77 |
| 56 | 54.0 ± 38.84 | 40.8 ± 20.44 | 32.5 ± 9.70 | 42.9 ± 15.45 |
| 90 | 93.8 ± 148.65 | 44.9 ± 24.33 | 36.5 ± 13.80 | 50.3 ± 13.56 |
| 104 | 57.3 ± 32.61 | 46.5 ± 25.45 | 36.0 ± 20.03 | 46.4 ± 20.39 |
| **Magnesium (mEq/L)** | | | | |
| -9 | 1.625 ± 0.104 | 1.700 ± 0.093 | 1.675 ± 0.104 | 1.638 ± 0.092 |
| 14 | 1.638 ± 0.119 | 1.638 ± 0.119 | 1.638 ± 0.130 | 1.575 ± 0.071* |
| 28 | 1.700 ± 0.107* | 1.700 ± 0.107 | 1.725 ± 0.116 | 1.613 ± 0.113 |
| 56 | 1.563 ± 0.106 | 1.638 ± 0.074 | 1.613 ± 0.125* | 1.575 ± 0.071* |
| 90 | 1.575 ± 0.128 | 1.625 ± 0.116* | 1.588 ± 0.113* | 1.638 ± 0.092 |
| 104 | 1.663 ± 0.106 | 1.675 ± 0.116 | 1.650 ± 0.160 | 1.625 ± 0.139 |
| **Sodium/potassium ratio** | | | | |
| -9 | 33.5 ± 1.41 | 30.8 ± 1.91 | 32.4 ± 2.67 | 33.5 ± 2.45 |
| 14 | 32.9 ± 3.44 | 33.1 ± 1.64* | 34.0 ± 2.27* | 34.3 ± 2.05 |
| 28 | 31.5 ± 2.67 | 31.9 ± 1.55 | 33.5 ± 2.56* | 33.9 ± 1.64 |
| 56 | 33.8 ± 1.98 | 32.9 ± 2.17* | 32.9 ± 2.42 | 33.9 ± 1.73 |
| 90 | 33.3 ± 2.25 | 32.0 ± 2.73 | 33.5 ± 2.00 | 34.9 ± 2.17* |
| 104 | 32.3 ± 1.58 | 31.3 ± 2.76 | 33.4 ± 1.85 | 33.6 ± 2.07 |
| **Phosphate (mg/dL)** | | | | |
| -9 | 4.800 ± 0.571 | 4.388 ± 0.824 | 4.238 ± 0.833 | 4.425 ± 0.891 |
| 14 | 4.113 ± 0.348* | 3.800 ± 0.635* | 3.975 ± 0.528 | 4.200 ± 0.540 |
| 28 | 4.163 ± 0.297* | 3.825 ± 0.580* | 3.688 ± 0.844* | 3.613 ± 0.636* |
| 56 | 4.013 ± 0.726* | 3.788 ± 0.624* | 3.838 ± 0.414 | 3.900 ± 0.685* |
| 90 | 4.088 ± 0.528* | 4.200 ± 0.472 | 3.900 ± 0.407 | 4.213 ± 0.497 |
| 104 | 4.325 ± 0.443* | 3.813 ± 0.482* | 3.788 ± 0.596* | 4.200 ± 0.504 |
| **Potassium (mEq/L)** | | | | |
| -9 | 4.375 ± 0.167 | 4.763 ± 0.302 | 4.575 ± 0.392 | 4.388 ± 0.275 |
| 14 | 4.500 ± 0.501 | 4.425 ± 0.183* | 4.325 ± 0.260* | 4.300 ± 0.239 |
| 28 | 4.663 ± 0.424 | 4.600 ± 0.200 | 4.413 ± 0.336 | 4.313 ± 0.164 |
| 56 | 4.338 ± 0.256 | 4.513 ± 0.285* | 4.475 ± 0.301 | 4.363 ± 0.233 |
| 90 | 4.463 ± 0.302 | 4.675 ± 0.315 | 4.463 ± 0.239 | 4.313 ± 0.309 |
| 104 | 4.538 ± 0.239 | 4.688 ± 0.380 | 4.400 ± 0.262* | 4.363 ± 0.267 |
| **Sodium (mEq/L)** | | | | |
| -9 | 146.6 ± 1.19 | 146.3 ± 1.28 | 146.8 ± 1.04 | 146.3 ± 1.67 |
| 14 | 146.0 ± 1.31 | 146.5 ± 1.93 | 146.6 ± 0.92 | 146.4 ± 1.30 |
| 28 | 146.3 ± 1.67 | 146.0 ± 1.07 | 146.9 ± 1.46 | 146.9 ± 1.36 |
| 56 | 146.4 ± 1.06 | 147.4 ± 1.41 | 147.5 ± 0.93 | 147.9 ± 1.36* |
| 90 | 148.0 ± 1.51* | 148.6 ± 1.85* | 150.0 ± 1.77* | 150.1 ± 1.96* |
| 104 | 147.1 ± 1.13 | 146.3 ± 2.12 | 146.6 ± 2.20 | 146.9 ± 0.64 |
| **Total bilirubin (mg/dL)** | | | | |
| -9 | 0.063 ± 0.052 | 0.038 ± 0.052 | 0.038 ± 0.052 | 0.025 ± 0.046 |
| 14 | 0.075 ± 0.046 | 0.025 ± 0.046 | 0.100 ± 0.000* | 0.025 ± 0.046 |
| 28 | 0.050 ± 0.053 | 0.063 ± 0.052 | 0.063 ± 0.052 | 0.050 ± 0.053 |
| 56 | 0.063 ± 0.052 | 0.000 ± 0.000* | 0.038 ± 0.052 | 0.000 ± 0.000 |
| 90 | 0.025 ± 0.046 | 0.000 ± 0.000* | 0.000 ± 0.000 | 0.013 ± 0.035 |
| 104 | 0.038 ± 0.052 | 0.050 ± 0.053 | 0.038 ± 0.052 | 0.025 ± 0.046 |
| **Total protein (g/dL)** | | | | |
| -9 | 5.875 ± 0.255 | 5.800 ± 0.214 | 5.875 ± 0.266 | 5.813 ± 0.264 |
| 14 | 5.713 ± 0.290 | 5.513 ± 0.242* | 5.688 ± 0.270* | 5.438 ± 0.177* |
| 28 | 5.850 ± 0.355 | 5.650 ± 0.298 | 5.763 ± 0.272 | 5.575 ± 0.205* |
| 56 | 5.775 ± 0.320 | 5.700 ± 0.396 | 5.763 ± 0.141 | 5.663 ± 0.245 |
| 90 | 5.938 ± 0.302 | 5.725 ± 0.354 | 5.863 ± 0.272 | 5.713 ± 0.360 |
| 104 | 5.950 ± 0.346 | 5.788 ± 0.340 | 5.750 ± 0.385 | 5.675 ± 0.423 |
| **Urea (mg/dL)** | | | | |
| -9 | 10.3 ± 1.75 | 10.0 ± 2.07 | 10.5 ± 2.56 | 11.6 ± 1.30 |
| 14 | 14.3 ± 2.19* | 12.8 ± 2.25* | 12.3 ± 1.83 | 11.6 ± 2.20 |
| 28 | 14.5 ± 2.20* | 13.4 ± 2.13* | 14.1 ± 1.36* | 14.1 ± 1.36* |
| 56 | 13.9 ± 2.64* | 12.1 ± 1.64* | 13.3 ± 2.71* | 13.5 ± 1.77* |
| 90 | 13.3 ± 2.31* | 12.5 ± 2.07* | 14.1 ± 1.96* | 13.5 ± 1.20* |
| 104 | 15.0 ± 2.33* | 13.5 ± 2.67* | 13.3 ± 2.19* | 12.8 ± 2.12 |

Data are reported as mean ± SD of serum chemistry parameters on each assessment day.

*Significantly different from baseline (P < .05).

µg = Microgram. CBD = Cannabidiol. CBDA = Cannabidiolic acid. CBG = Cannabigerol. dL = Deciliter. g = Gram. L = Liter. MCT = Medium chain triglyceride. mEq = Milliequivalents. mg = Milligram. U = Units.

**Supplementary Table 5 -** Summary of clinical chemistry parameters with statistically significant differences between treatment and control for healthy beagles treated orally with MCT oil (Control; n=8) or 5 mg/kg bw/day of CBD (n=8), CBD + CBG (n=8) or CBD + CBDA (n=8) for 90 days, followed by 14 days without dosing.

|  | **Study Day** | | | | |
| --- | --- | --- | --- | --- | --- |
| **Parameter** | 14 | 28 | 56 | 90 | 104 |
| Albumin/globulin ratio |  |  | 2↓-*,3↓-*,4↓-* |  |  |
| Albumin (g/dL) | 4↓+* |  | 2↓+*,3↓+*,4↓+* |  | 3↓+*,4↓+* |
| ALP (U/L) |  | 2↑+* | 2↑+*, 4↑+* | 2↑+* |  |
| ALT (U/L) |  | 3↓+* | 3↓+* | 2↓-*,4↓+* | 3↓+* |
| Amylase (U/L) |  |  |  |  | 4↓-* |
| Anion gap (mEq/L) | 2↓- |  |  |  |  |
| Bicarbonate (mEq/L) |  |  |  |  | 4↑- |
| Chloride (mEq/L) |  |  | 4↑±* |  |  |
| Cholesterol (mg/dL) |  |  |  | 2↑-* |  |
| Creatine kinase (U/L) |  |  |  |  | 3↑+ |
| FE saturation (%) |  |  |  | 2↑+ |  |
| GGT (U/L) |  | 2↓-* |  |  |  |
| Hemolytic Index |  | 2↑-, 3↑-, 4↑- |  |  |  |
| Iron (µg/dL) |  | 4↑- |  |  |  |
| LDH (U/L) | 3↑- |  |  |  |  |
| Magnesium (mEq/L) |  | 4↓-* |  |  |  |
| Phosphate (mg/dL) |  |  |  | 2↓- |  |
| Potassium (mEq/L) | 2↓+*, 3↓+* | 2↓-*, 3↓-*, 4↓-* |  |  | 3↓+* |
| Sodium (mEq/L) |  |  | 4↑+* | 3↑+, 4↑+ |  |
| Sodium/potassium ratio | 2↑+*, 3↑+* | 2↑-*, 3↑-*, 4↑-* |  |  | 3↑-* |
| Total protein (g/dL) | 4↓+ |  |  |  |  |
| Urea Nitrogen (mg/dL) | 4↑-** |  |  |  | 4↑- |
| All data presented are statistically significant comparisons between the mean change from baseline (CFB) of the treatment compared to control for the study day. No statistically significant differences between treatment CFB and control CFB were found for AST, Calcium, Creatinine, bilirubin (direct, indirect or total), globulin, glucose, lipase, lipemia, or total iron binding capacity.  Number indicates treatment: 2=CBD, 3=CBD + CBG, 4=CBD + CBDA.  "+" indicates CFB for treatment is larger in absolute magnitude than control.  "-" indicates CFB for treatment is smaller in absolute magnitude than control.  “±” indicates equal absolute magnitude in opposite directions (one incidence).  ↑ indicates CFB in treatment group showed an increase in the parameter value from baseline (positive CFB).  ↓ indicates CFB in treatment group showed a decrease in the parameter value from baseline (negative CFB).  * indicates treatment mean CFB was the opposite direction from the control (i.e., treatment mean CFB showed an increase in the parameter value and control mean CFB showed a decrease in the parameter value, or vice versa).  ** CFB in treatment group was 0 (one incidence).  µg = Microgram. CBD = Cannabidiol. CBDA = Cannabidiolic acid. CBG = Cannabigerol. dL = Deciliter. g = Gram. L = Liter. mg = Milligram. mEq = Milliequivalent. U = Units. | | | | | |

**Supplementary Table 6 -** Mean coagulation results for healthy beagles treated orally with MCT oil (control; n=8) or 5 mg/kg bw/day of CBD (n=8), CBD + CBG (n=8) or CBD + CBDA (n=8) for 90 days, followed by 14 days without dosing. Baseline is study day -9 for the purposes of statistical comparison.

| **Study day** | **Control** | **CBD** | **CBD + CBG** | **CBD + CBDA** |
| --- | --- | --- | --- | --- |
| **Fibrinogen (mg/dL)** | | | | |
| -9 | 247.0 ± 57.50 | 260.8 ± 65.30 | 278.1 ± 33.41 | 266.8 ± 50.12 |
| 14 | 212.8 ± 27.00 | 340.0 ± 196.42 | 247.8 ± 21.53 | 279.9 ± 46.92 |
| 28 | 228.1 ± 45.85 | 298.3 ± 82.08 | 260.1 ± 14.84 | 314.5 ± 100.45* |
| 56 | 192.6 ± 14.52 | 276.1 ± 36.98 | 280.9 ± 35.75 | 279.0 ± 42.17 |
| 90 | 377.3 ± 208.73* | 411.4 ± 105.20* | 347.3 ± 45.50* | 334.5 ± 75.82* |
| 104 | 293.3 ± 26.46 | 344.3 ± 74.31 | 352.4 ± 93.54* | 322.1 ± 48.60* |
| **Prothrombin time (s)** | | | | |
| -9 | 11.20 ± 0.417 | 11.41 ± 0.613 | 12.11 ± 1.905 | 11.43 ± 0.420 |
| 14 | 12.65 ± 0.321* | 11.74 ± 0.863 | 11.78 ± 1.422 | 10.60 ± 0.807* |
| 28 | 12.53 ± 0.191* | 11.98 ± 0.684 | 12.93 ± 2.264* | 11.31 ± 0.304 |
| 56 | 12.56 ± 0.463* | 11.10 ± 1.183 | 11.98 ± 1.950 | 10.84 ± 0.381* |
| 90 | 11.65 ± 0.227* | 11.64 ± 0.652 | 12.36 ± 2.550 | 11.46 ± 0.550 |
| 104 | 11.71 ± 0.314* | 11.48 ± 0.800 | 12.03 ± 2.242 | 10.86 ± 0.614* |
| **Activated partial thromboplastin time (s)** | | | | |
| -9 | 12.71 ± 1.363 | 12.95 ± 1.072 | 12.19 ± 0.572 | 12.38 ± 1.341 |
| 14 | 12.19 ± 0.942 | 12.26 ± 1.023* | 10.86 ± 0.953* | 11.63 ± 0.358* |
| 28 | 12.33 ± 1.055 | 11.98 ± 0.826* | 11.59 ± 0.601 | 11.66 ± 0.498* |
| 56 | 12.40 ± 1.102 | 12.24 ± 0.802* | 11.75 ± 0.778 | 11.63 ± 0.692* |
| 90 | 13.70 ± 0.935* | 13.46 ± 0.819 | 13.23 ± 0.959* | 12.95 ± 0.941 |
| 104 | 12.81 ± 1.448 | 12.95 ± 1.039 | 12.46 ± 0.936 | 11.91 ± 0.775 |

Data are reported as mean ± SD of coagulation parameters on each assessment day.

*Significantly different from baseline (row) (P < .05).

CBD = Cannabidiol. CBDA = Cannabidiolic acid. CBG = Cannabigerol. dL = Deciliter. MCT = Medium chain triglycerides.

**Supplementary Table 7** - Summary of mean urine results for healthy beagles treated with MCT oil (control; n=6) or 5 mg/kg bw/day of CBD (n=6), CBD + CBG (n=6) or CBD + CBDA for 90 days, followed by 14 days without dosing. Baseline is study day -8 or -7 for the purposes of statistical comparison.

| **Study day** | **Control** | **CBD** | **CBD + CBG** | **CBD + CBDA** |
| --- | --- | --- | --- | --- |
| **Specific gravity** | | | | |
| -8/-7 | 1.0353 ± 0.0221 | 1.0423 ± 0.0161 | 1.0243 ± 0.0187 | 1.0290 ± 0.0133 |
| 28 | 1.0273 ± 0.0172 | 1.0374 ± 0.0155 | 1.0458 ± 0.0087 | 1.0234 ± 0.0105 |
| 90 | 1.0386 ± 0.0111 | 1.0448 ± 0.0100 | 1.0299 ± 0.0111 | 1.0338 ± 0.0152 |
| 104 | 1.0281 ± 0.0116 | 1.0381 ± 0.0170 | 1.0359 ± 0.0176 | 1.0275 ± 0.0180 |
| **pH** | | | | |
| -8/-7 | 6.94 ± 1.016 | 7.25 ± 1.000 | 7.13 ± 0.954 | 6.44 ± 0.563 |
| 28 | 7.81 ± 0.884 | 6.31 ± 0.372 | 6.67 ± 0.983 | 7.94 ± 1.050 |
| 90 | 7.44 ± 1.116 | 6.79 ± 1.035 | 8.13 ± 0.916 | 7.88 ± 1.188 |
| 104 | 7.75 ± 0.845 | 8.19 ± 0.753 | 7.31 ±1.223 | 7.88 ± 0.916 |

Data are reported as mean ± SD.

CBD = Cannabidiol. CBDA = Cannabidiolic acid. CBG = Cannabigerol. MCT = Medium chain triglycerides.
